# Shikimic acid protects skin cells from UV-induced senescence through activation of the NAD+-dependent deacetylase SIRT1

**DOI:** 10.18632/aging.203010

**Published:** 2021-04-26

**Authors:** Alfredo Martínez-Gutiérrez, Irene Fernández-Duran, Anna Marazuela-Duque, Nicolás G. Simonet, Ibraheem Yousef, Immaculada Martínez-Rovira, Josefina Martínez-Hoyos, Alejandro Vaquero

**Affiliations:** 1 Chromatin Biology Laboratory, Josep Carreras Leukaemia Research Institute (IJC), Barcelona 08916, Badalona, Spain; 2 Chromatin Biology Laboratory, Cancer Epigenetics and Biology Program (PEBC), Bellvitge Biomedical Research Institute (IDIBELL), Barcelona 08908, Spain; 3 Mesostetic Pharma Group, Barcelona 08840, Viladecans, Spain; 4 MIRAS Beamline, ALBA-CELLS Synchrotron, Barcelona 08290, Cerdanyola del Vallès, Spain; 5 Ionizing Radiation Research Group, Physics Department, Universitat Autònoma de Barcelona (UAB), Barcelona 08193, Cerdanyola del Vallès, Spain

**Keywords:** SIRT1, senescence, human dermal fibroblasts, UV irradiation, shikimic acid

## Abstract

UV radiation is one of the main contributors to skin photoaging by promoting the accumulation of cellular senescence, which in turn induces a proinflammatory and tissue-degrading state that favors skin aging. The members of the sirtuin family of NAD^+^-dependent enzymes play an anti-senescence role and their activation suggests a promising approach for preventing UV-induced senescence in the treatment of skin aging. A two-step screening designed to identify compounds able to protect cells from UV-induced senescence through sirtuin activation identified shikimic acid (SA), a metabolic intermediate in many organisms, as a bona-fide candidate. The protective effects of SA against senescence were dependent on specific activation of SIRT1 as the effect was abrogated by the SIRT1 inhibitor EX-527. Upon UV irradiation SA induced S-phase accumulation and a decrease in *p16^INK4A^* expression but did not protect against DNA damage or increased polyploidies. In contrast, SA reverted misfolded protein accumulation upon senescence, an effect that was abrogated by EX-527. Consistently, SA induced an increase in the levels of the chaperone BiP, resulting in a downregulation of unfolded protein response (UPR) signaling and UPR-dependent autophagy, avoiding their abnormal hyperactivation during senescence. SA did not directly activate SIRT1 *in vitro*, suggesting that SIRT1 is a downstream effector of SA signaling specifically in the response to cellular senescence. Our study not only uncovers a shikimic acid/SIRT1 signaling pathway that prevents cellular senescence, but also reinforces the role of sirtuins as key regulators of cell proteostasis.

## INTRODUCTION

Skin aging is defined as the degenerative process by which structural and physiological alterations cause the impairment of skin biological functions [1–3]. It can be classified as intrinsic or extrinsic, depending on the origin of the type of stress that causes aging. Intrinsic skin aging, also known as chronological aging, depends on the passage of time and is influenced by the genetic background and hormonal levels. In contrast, extrinsic skin aging, or photoaging, is caused by external stressors such as UV light, pollution, or tobacco smoke [1]. UV light is the main contributor to photoaging [4–6], and may be divided into UVB, which reaches the epidermis and the upper dermis, and UVA, which involves longer UV wavelength radiation able to penetrate into the dermis.

The development of skin aging is associated with several changes at the molecular level, including the accumulation of DNA damage, genome instability, epigenetic dysregulation, extracellular matrix degradation, mitochondrial dysfunction, inflammation, loss of proteostasis, ER-stress and autophagy dysfunction. Many of these alterations are directly associated with cellular senescence, one of the hallmarks of skin aging. Cellular senescence is a state in which cells enter permanent cell cycle arrest and acquire a variety of phenotypic changes related to gene expression, cellular functions and morphology [7]. This process is normally induced after exposure to mild, chronic damage, such as the exposure of the skin to UV rays from sunlight. Other known inducers of cellular senescence include telomere shortening, oncogene activation, genomic damage and mitochondrial dysfunction [8, 9]. Upon DNA damage, several pathways, including p53, p38-MAPK, NF-κβ and mTOR pathways, are activated to establish and maintain cellular senescence [10–12]. These pathways are then involved in cell cycle arrest and induction of a pro-inflammatory state (known as the senescence-associated secretory phenotype, SASP), among other processes, which is one of the major features of cell senescence. The SASP creates an inflammatory environment that favors the degradation of extracellular matrix proteins and promotes senescence in the surrounding cells, which contribute to tissue damage, aging and tumorigenesis. Senescent cells are also characterized by other features, which are routinely used as biomarkers to identify senescence *in vitro* and *in vivo*. These features include large and flattened cell morphology, induction of senescence-associated β-galactosidase (SA-β-Gal) activity, chromatin rearrangement, increased glycolysis, expression of immunogenic-related proteins, activation of pro-survival pathways, DNA damage, loss of proteostasis and mitochondrial damage [13]. Of these, increased levels of SA-β-Gal activity is currently the most widely accepted biomarker to identify senescence cells. However, as none of the aforementioned senescence features is specific to senescence, the detection of more than one marker is required to confirm the senescent state [14].

Of the factors associated with this process, sirtuins play essential roles by protecting against cellular senescence and aging [15]. Sirtuins are a family of NAD^+^-dependent deacetylases conserved throughout evolution, from bacteria to humans. These proteins regulate the stress response of the cell through their involvement in many cellular processes, including chromatin regulation, DNA repair, metabolism and inflammation. There are seven sirtuins in humans, named SIRT1-SIRT7, which are located in different cellular compartments and perform specific and redundant functions [16]. SIRT1, SIRT2, SIRT3, SIRT6, and to a lesser extent SIRT7, are known to play a role in senescence [17].

Given the increasing evidence linking sirtuins, and SIRT1 in particular, to skin senescence and aging, the modulation of sirtuin activity in this functional context is a promising approach for delaying the development of UV-induced skin aging. In this sense, a number of SIRT1 specific activators have been shown to prevent senescence, inhibit inflammation and promote wound healing in dermal fibroblasts [18–20]. Moreover, some of them are currently used in the treatment of psoriasis or are being evaluated clinically for this purpose [21]. However, although several sirtuin activators have been described, the few that are of relevance in dermatology have limited efficacy and skin penetration [22, 23]. Therefore, the identification of novel sirtuin activators in skin is potentially very significant for the prevention of skin aging.

In the work presented here, we have performed a screening to identify novel molecules that induce sirtuin activation and protect against UV-induced cellular senescence. We selected the most promising compounds from the screening and characterized in greater detail their effect on senescence, sirtuin activation and targets in order to describe novel compounds that are effective at prevent skin photoaging.

## RESULTS

In our efforts to identify novel factors that could prevent skin cell senescence, we developed a screening process to identify new modulators of the protective role of sirtuins in UV-induced senescence. For this purpose, we established a culture of primary human dermal fibroblasts (HDFs). For the screening, we selected 30 compounds that had previously been shown to regulate skin senescence and/or aging or had been linked to sirtuin function (Table 1, left). Our first step in the screening was to establish the optimal concentrations of these compounds in the case of HDFs. For this purpose, we calculated for each compound the lethal dose 50 (LD50) and the maximal non-cytotoxic dose conditions (WST-1 cell viability test), which we used to perform the screening (Table 1, right). Once established, we took these parameters into account to decide the specific working range of concentrations for each compound.

**Table 1:** List of compounds chosen to be tested.

**Compound**	**Target**	**Biological process**	**Species**	**References**	**LD_50_ (mM)**	**Max. non-cyt. doses (mM)**
Resveratrol	Sir2, SIRT1	SIRT1 activation	Y, H	[24–26]	0.173	0.05
Quercetin	Sir2, SIRT1	SIRT1 activation	Y, H	[24–26]	0.604	0.25
Dexpanthenol	-	SH, WH	H	[27]	262.48	100
Allantoin	AMPK	GM	M	[28]	100	50
Idebenone	BAX/Bcl-2 ratio	SEN	H	[29]	0.021	0.01
Ferulic acid	FOXO3a	AG	M	[30]	16.5	7.5
Lipoic acid	SIRT1-AMPK	LM	M	[31]	34.69	1
Gallic acid	AMPK-SIRT1	AP, GM, LM	H, M	[32]	0.179	0.1
Taurine	-	SEN	M	[33]	396.58	150
Salicylic acid	AMPK	LM	H, M	[34]	20.88	1
Shikimic acid	Nrf2-NF-κβ	OS	M	[35]	58.44	25
Ectoine	-	PA	H	[36]	359.12	300
3,4-dihydroxybenzaldehyde	JMJD2a	L	D	[37]	1.08	0.1
Andrographolide	PI3K/Akt/Nrf2 PI3K/Akt/AP-1	INF	H	[38]	0.124	0.01
Kinetin	-	PA	H	[39]	1.835	0.5
Zeatin	-	AG	H	[40]	2.462	1
Carnosine		SEN	H	[41]	85.46	25
Damascenone		PA	M	[42]	0.121	0.05
Betaine	NF-κβ	INF	R	[43]	507.81	50
Pyridoxine	-	OS	M	[44]	16.92	5
Verbascoside	-	PA	H	[45]	0.122	0.05
Hamamelitannin	-	PA	M	[46]	0.247	0.1
Phloretin	SOD-Sir2	RL	Y	[47]	0.166	0.05
Vanillin	ATM-p53-JNK	UVDD	H	[48]	5.912	1
Sesamol	-	PA	M	[49]	4.358	0.5
Salicyladehyde thiosemicarbazone	-	OS	H	[50]	4.175	0.5
Syringic acid	NF-κβ-TLR4	INF, LM	M	[51]	3.65	1
Sclareol	-	INF	M	[52]	0.04	0.02
Gentiopicrin	p38-ERK-JNK	INF	R	[53]	13.825	2.5
Irisflorentin	ERK1/2-AP-1	INF	M	[54]	0.049	0.01

Left columns show the molecular targets, biological processes and species. Right columns show the calculated LD50 and the maximum non-cytotoxic dose (mM) in our cell viability assay for each compound. Aging (AG), autophagy (AP), glucose metabolism (GM), inflammation (INF), lifespan (L), lipid metabolism (LM), oxidative stress (OS), photoaging (PA), senescence (SEN), skin hydration (SH), UV-induced DNA damage (UVDD), wound healing (WH). Species: chicken (C), *C. elegans* (CE), *D. melanogaster* (D), hamster (HA), human (H), mouse (M), rat (R), yeast (Y).

### Shikimic acid is a senescence inhibitor that promotes sirtuin activity

We performed the screening by merging two sequential approaches. The first one was a cellular assay in which we tested the ability of these compounds to promote a decrease in sirtuins targets that would indicate an increased sirtuin activity. The second one was a senescence assay in which we determined the effect of the compounds on cellular senescence induced by UV irradiation.

**Figure 1:**
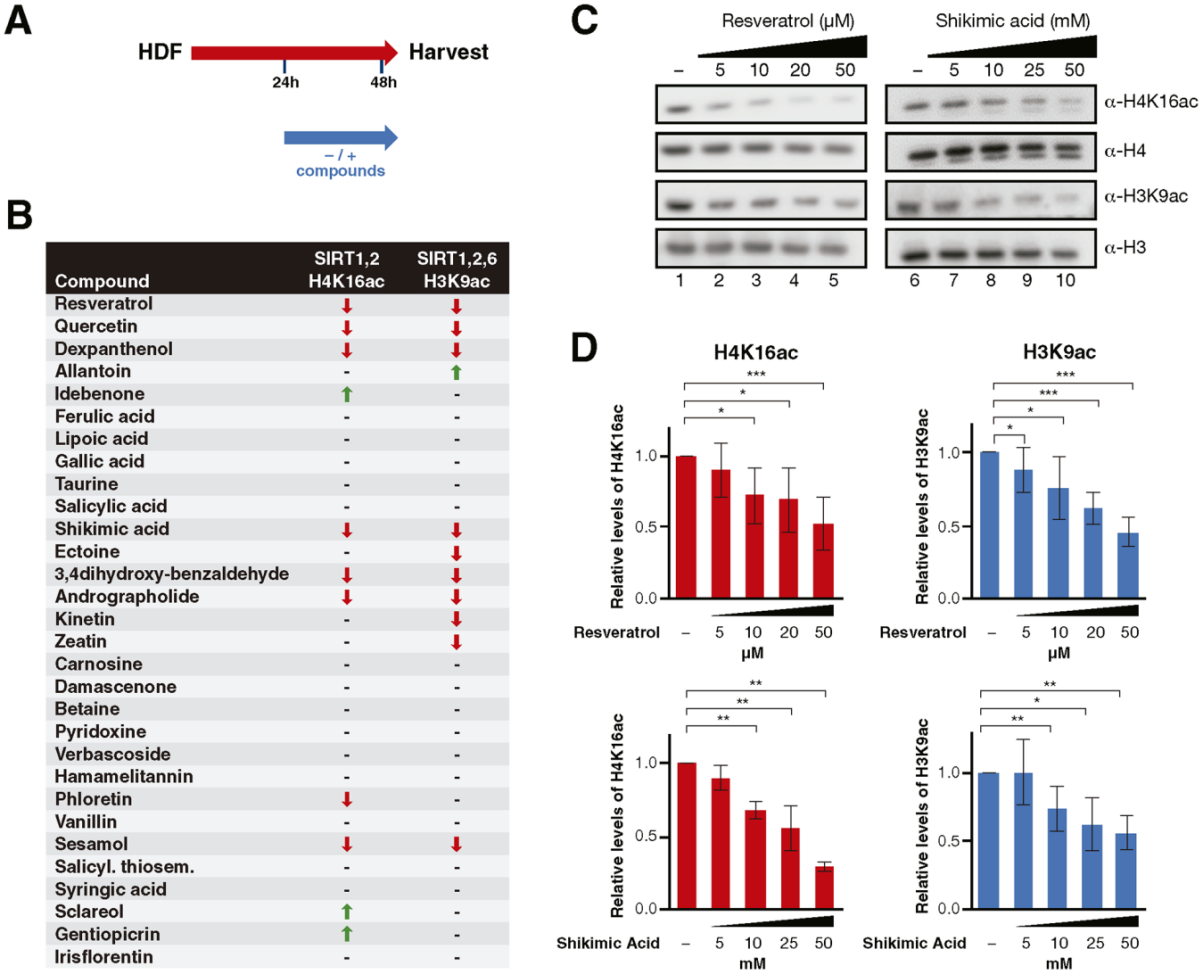
(**A**) Schematic representation of the compound testing for sirtuin activity evaluation in HDF. Cells were treated with or without the compounds for 24h and harvested for analysis. (**B**) Sirtuin activity assay results including H4K16 and H3K9 acetylation for cells treated with each compound. The results are shown by arrows that indicate increase/decrease (↑, ↓) and hyphens that indicate no effect (-). (**C**) Western Blot analysis and (**D**) quantification of H4K16ac and H3K9ac levels in cells treated with Resveratrol or Shikimic acid at the indicated concentrations. H4K16 and H3K9 levels were normalized to H4 and H3 levels respectively. Student T-test, *p<0.05, **p<0.01, ***p<0.001.

In the first approach, we tested the ability of the 30 compounds to activate sirtuin activity, for which purpose, we treated the cells for 24 h and monitored the effect of the compounds on two of the best characterized sirtuin targets, the histone marks H4K16ac (the target of SIRT1 and SIRT2) and H3K9ac (the target of SIRT1, SIRT2 and SIRT6) (Figure 1B). In these studies we included the well described SIRT1 activator resveratrol [18], and quercetin, which has been shown to activate or inhibit SIRT1 in different functional contexts [26, 55]. Seven compounds (Resveratrol, Quercetin, Dexpanthenol, Shikimic acid, 3,4-dihydroxy-benzaldehyde, Andrographolide, Sesamol) induced a similar effect on both targets (Figure 1B–1D and Supplementary Figure 1). Three compounds reduced the levels of H3K9ac only (Ectoine, Kinetin, Zeatin), and one compound (Phloretin) had an effect solely on H4K16ac (Supplementary Figure 1).

**Figure 2:**
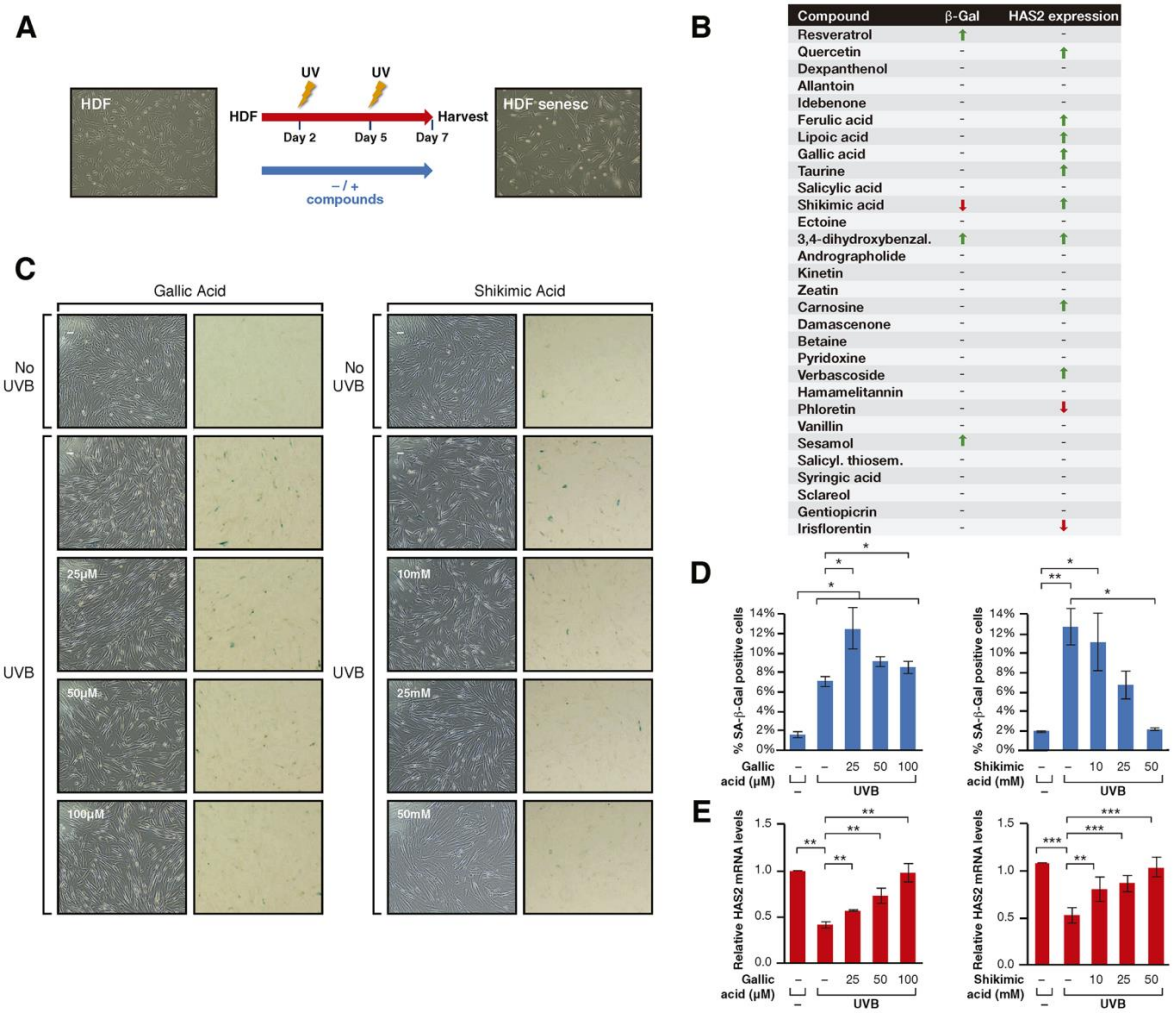
(**A**) Schematic representation of the protocol for senescence induction using UVB irradiation. Cells were treated -/+ the compounds from day 1 to 7 after seeding, irradiated with UVB on days 2 and 5 and harvested for analysis on day 7. (**B**) Senescence assay results including β-Gal staining and *HAS2* gene expression analysis for cells treated with each compound. The results are shown by arrows that indicate increase/decrease (↑, ↓) and hyphens that indicate no effect (-). (**C**) Phase-contrast (left column) and bright field (right column) microscope images of SA-β-Gal staining showing non-irradiated cells, UVB-irradiated cells non-treated and treated with Gallic acid or Shikimic acid at the indicated concentrations. (**D**) Percentage of SA-β-Gal positive cells and (**E**) Relative *HAS2* mRNA levels in non-irradiated cells, UVB-irradiated cells and UVB-irradiated cells treated with Gallic acid or Shikimic acid at the indicated concentrations. Student T-test, *p<0.05, **p<0.01, ***p<0.001.

In the second approach, we established a method for inducing senescence in these cells with 2 cycles of UVB irradiation 72h apart (Figure 2A). The use of UVA to induce senescence was discarded due to its considerably longer time requirement, being unsuitable to screen 30 compounds within a reasonable period. Cells were treated with these compounds throughout the duration of the assay and cellular senescence was monitored with two markers: accumulation of SA-β-galactosidase (SA-β-Gal) activity, and the decrease in expression of *HAS2* (hyaluronic acid synthase 2), a previously reported marker of skin senescence [56]. Our senescence analysis considered positive candidates to be only those compounds capable of inducing changes in both markers (Figure 2B). This first stage showed that, of the 30 compounds, only shikimic acid (SA), one of the positive candidates identified by the first approach, prevented the development of senescence, reflected by both a significant decrease in SA-β-Gal accumulation and an increase in *HAS2* expression (Figure 2C–2E, Supplementary Figures 2, 3). Interestingly, SIRT1 reported activators Resveratrol and Quercetin either only promoted *HAS2* upregulation (Quercetin) or increased SA-β-Gal staining (Resveratrol) (Figure 2B and Supplementary Figure 2). This observation was in part explained by the fact that the identified range of concentrations able to activate Sirtuin activity in the first stage (Figure 1), induced cell death in the second stage of the screening. Moreover, the other four compounds (Dexpanthenol, 3,4-dihydroxy-benzaldehyde, Andrographolide, Sesamol) that induced the deacetylation of sirtuin targets in the previous screening (Figure 1B), did not prevent cellular senescence. Although six other compounds induced *HAS2* expression (Ferulic and Lipoic acids, Taurine, Carnosine and Verbascoside), they did not change the level of SA-β-Gal staining (Figure 2B). Overall, the combined result of the two approaches identified SA as being the only *bona fide* candidate for preventing cellular senescence through sirtuin activation.

### SA prevents UV-induced senescence through SIRT1

**Figure 3:**
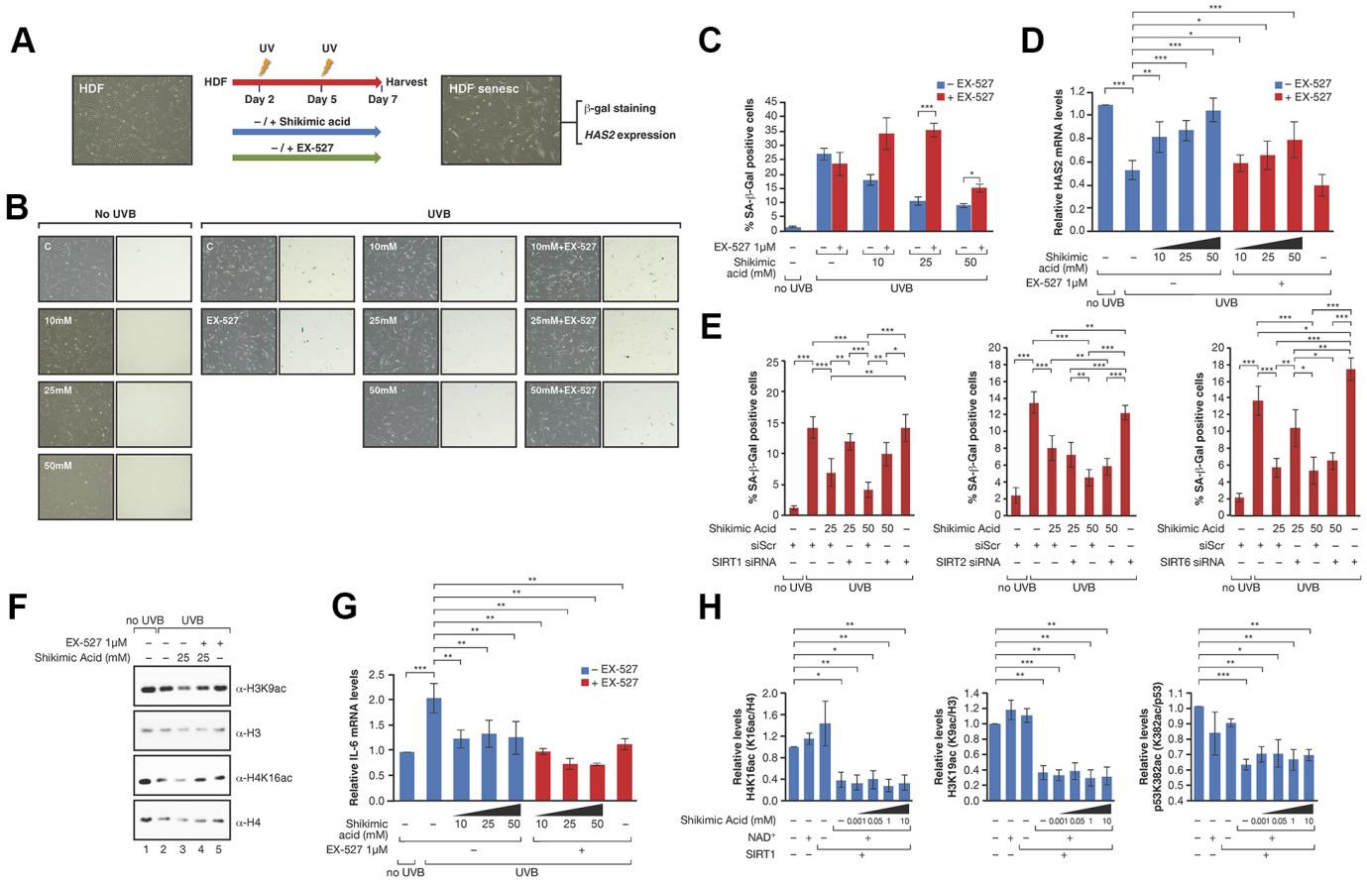
(**A**) Schematic representation of the protocol for senescence induction using UVB irradiation and study of EX527 involvement in the effect of Shikimic acid. Cells were treated with or without shikimic acid -/+ EX-527 from day 1 until day 7 after seeding, irradiated with UVB on days 2 and 5 and harvested for analysis on day 7. (**B**) Phase-contrast (left column) and bright field (right column) microscope images of β-Gal staining showing non-irradiated cells non-treated and treated with shikimic acid (10, 25 and 50 mM), UVB-irradiated cells non-treated and treated with shikimic acid (10, 25 and 50 mM), shikimic acid (10, 25 and 50 mM) + EX-527 1 μM and EX-527 1 μM. (**C**) Percentage of SA-β-Gal positive cells and (**D**) relative *HAS2* mRNA levels in non-irradiated cells non-treated and treated with Shikimic acid (10, 25 and 50 mM), UVB-irradiated cells non-treated and treated with Shikimic acid (10, 25 and 50 mM), Shikimic acid (10, 25 and 50 mM) + EX-527 1 μM and EX-527 1 μM. (**E**) Percentage of SA-β-Gal as in (**C**) of SA 25 or 50mM upon siRNA mediated downregulation of SIRT1, SIRT2 or SIRT6. siScr: scramble siRNA. Student T-test, *p<0.05, **p<0.01, ***p<0.001. (**F**) Levels of H3K9ac/H3 and H4K16ac/H4 under UV-induced senescence in HFD incubated with -/+25mM SA -/+1μM EX-527. (**G**) IL-6 mRNA levels of the assays as in (**D**). (**H**) The activity of FLAG-SIRT1 purified from HEK293 cells was tested in *in vitro* deacetylation assays -/+ NAD^+^ -/+ Shikimic acid (1 μM-10mM) in presence of hyperacetylated core histones. SIRT1 activity was then evaluated by analyzing acetylation of H3K9, H4K16 and p53K382 by Western blot. Student T-test, *p<0.05, **p<0.01, ***p<0.001.

Next, we set out to understand the role of SA in this context. The decreased levels of both H4K16ac and H3K9ac pointed to the involvement of SIRT1, since it is the only one of these sirtuins that efficiently targets both marks. To confirm this involvement, we pharmacologically inhibited SIRT1, SIRT2 and SIRT6 with the compounds EX-527, AGK2 and OSS128167, respectively, and tested the ability of SA to prevent senescence (Figure 3A, 3B and Supplementary Figure 4). The working conditions were chosen either based on previous reports (EX-527, OSS128167) [57, 58] or determined experimentally to avoid excessive cell death (AGK2, data not shown). In the case of EX-527, our working concentration was 1μM, as it has been shown to inhibit SIRT1 specifically (IC50 100nM) in contrast to the other two described targets SIRT2 (IC50 19μM) and SIRT3 (IC50 40μM) [57]. As expected, treatment of EX-527 significantly inhibited the protective effect of SA on senescence at the level of SA-β-Gal and *HAS2* expression (Figure 3C, 3D), while AGK2 and OSS128167 produced no clear effect (Supplementary Figure 4A, 4B). Confirming these observations, siRNA-mediated downregulation of SIRT1 reverted the anti-senescent effect of SA 25mM and 50mM measured by SA-β-Gal levels. In contrast, downregulation of SIRT2 did not have any effect while SIRT6 downregulation induced by itself a SA-independent increase in the levels of senescence (Figure 3E).

In agreement with a SA-dependent modulation of SIRT1 activity, SA 25mM induced increased deacetylation of H3K9ac and H4K16ac under UV conditions in these cells while EX-527 reverted this effect (Figure 3F). We discounted the possibility that the effect of SA was due to an increase in SIRT1 levels, since we did not observe any significant correlation with gene expression and protein levels (Supplementary Figure 5A–5C). Interestingly, SA induced a reduction in the levels of expression of the inflammatory cytokine IL-6 (Figure 3G), previously shown to be regulated by SIRT1 [59]. Moreover, this decrease was further enhanced by EX-527, indicating that the anti-inflammatory role of SA is independent of SIRT1. This suggested that SA may not be a direct regulator of SIRT1. Consistently, SA did not alter SIRT1 deacetylation activity *in vitro* towards three different SIRT1 targets, H3K9ac, H4K16ac and p53K382ac (Figure 3H and Supplementary Figure 5D).

Overall, these results suggested that SIRT1 is an indirect downstream target of shikimic acid in this functional context.

### The protective effect of SA in HDF cellular senescence is not related to genome stability or cell death

**Figure 4:**
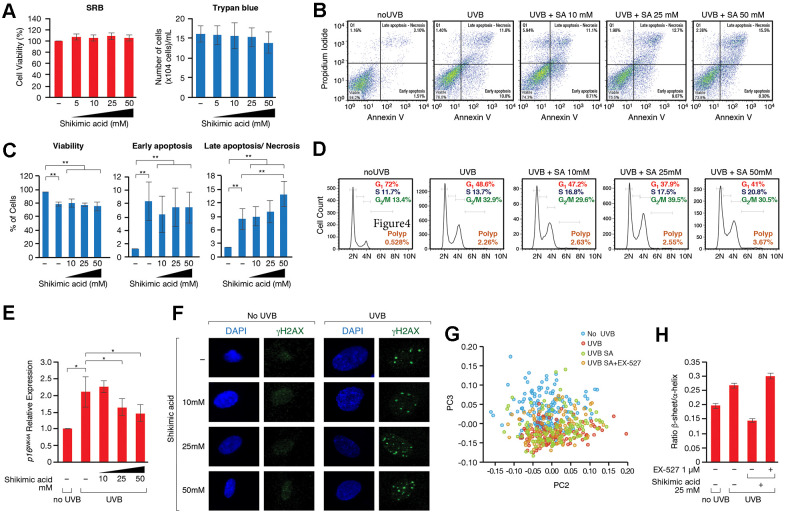
(**A**) SRB and Trypan blue cell viability assays performed in cells treated with or without Shikimic acid (5, 10, 25 and 50 mM) for 24h. (**B**) Apoptosis detection using the Annexin V-FITC/PI double staining followed by flow cytometry in non-irradiated and non-treated cells, irradiated and non-treated cells and irradiated cells treated with Shikimic acid (10, 25 and 50 mM). (**C**) Representation and statistical analysis of the percentage of apoptotic cells in each of the conditions analyzed. (**D**) Cell cycle analysis using PI staining followed by flow cytometry. (**E**) Relative *p16^INK4a^* mRNA levels monitored by qPCR in the indicated conditions. Student T-test, *p<0.05. (**F**) Immunofluorescence of γH2AX in non-irradiated and non-treated cells, irradiated and non-treated cells and irradiated cells treated with Shikimic acid (10, 25 and 50 mM). (**G**) Principal component analysis (PCA) on Savitzky–Golay second derivatized spectra in the fingerprint region (1800–950 cm−1) for non-irradiated cells (No UVB), irradiated non-treated cells (UVB), irradiated cells treated with Shikimic acid 25 mM (UVB SA) and irradiated cells treated with Shikimic acid 25 mM plus EX-527 1 μM (UVB SA+EX-527). Student T-test, *p<0.05, **p<0.01, ***p<0.001. (**H**) Beta-sheet to alpha-helix ratio obtained by curve-fitting analysis of the amide I band (1700-1600 cm-1).

Our next step was to understand the effect of SA in these HDF cells. We discounted the possibility that the effect was due to increased cell death as the SRB/trypan blue assay revealed no differences in cytotoxicity (Figure 4A), and annexin V assays showed very mild and mostly insignificant levels of early apoptosis in UVB-irradiated cells upon exposure to SA (Figure 4B, 4C). SA did not alter the cell cycle in the absence of UVB irradiation (Supplementary Figure 6A). While UVB induced an accumulation of cells in G_2_/M and a reduction in the abundance of cells in G_1_, SA induced a progressive increase in the number of cells in S-phase that was associated with a mild decrease in G_1_ and in G_2_/M (Figure 4D and Supplementary Figure 6B). This observation suggests that SA could participate in cell cycle checkpoint regulation, which was further supported by the downregulation of the cell cycle regulator *p16* observed upon SA treatment (Figure 4E). In contrast, we did not observe any clear effect on the levels of the cell cycle regulator, *p21* (Supplementary Figure 6C). These results suggest that SA exerts its protective function by specifically impeding the de-repression of the INK4/ARF locus. Indeed, activated SIRT1 has been suggested to be able to regulate p16 expression through epigenetic and genetic mechanisms [60], although how SIRT1 regulates p16 expression during cellular senescence remains to be fully elucidated. Consistently, incubation with SA did not alter the accumulation of DNA damage or the frequency of polyploidies induced by UVB irradiation, nor did it have any effect in the absence of the stress (Figure 4D–4F). Overall, this indicates that the effect of SA in senescence favors the entry of cells in S-phase, probably overcoming the G_1_/S checkpoint, but is unrelated to the control of genome integrity.

### SA regulates cell proteostasis through a SIRT1-dependent mechanism

**Figure 5:**
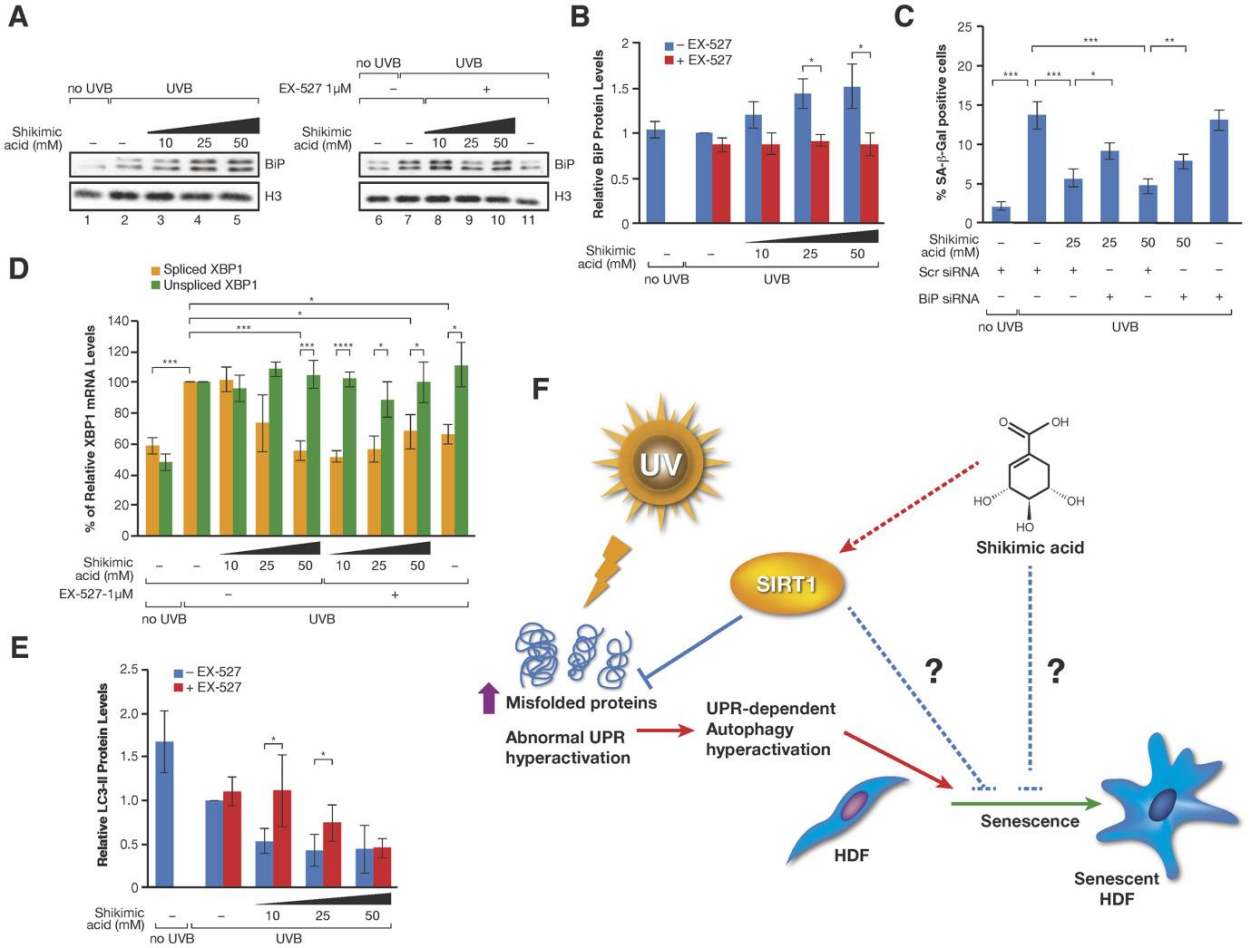
(**A**) Western-blot of BiP proteins levels in UVB or non UVB irradiated cells treated with SA at the indicated concentrations in presence or absence of EX-527 1 μM. (**B**) Quantification of n=3 experiments as (**A**). The levels of BiP are normalized by the loading control (histone H3) and represented related to the conditions in UVB irradiated cells in absence of SA and EX-527. Student T-test, *p<0.05. (**C**) Percentage of SA-β-Gal in assays performed as in Figure 3C. (**D**) Levels of spliced and unspliced XBP1 mRNA monitored by qPCR analysis in cells treated in the indicated conditions in presence or absence of SA and/or EX-527. The levels were normalized by internal controls and represented in each case related to the conditions in UVB irradiated cells in absence of SA and EX-527. Student T-test, *p<0.05, **p<0.01, ***p<0.001. (**E**) LC3-II protein levels in experiments like in (**A**). The levels of LC3-II are normalized by the loading control (tubulin) and represented related to the conditions in UVB irradiated cells in absence of SA and EX-527. Student T-test, *p<0.05. (**F**) Proposed Model for the effect of SA on senescence through SIRT1. Other possible unexplored connections between SA and senescence are indicated by blue broken lines.

Given the direct role of SA in metabolism, and particularly in aminoacid biosynthesis in many organisms, we considered the possibility that SA might regulate the recycling of unfolded or damaged proteins. In this sense, the unfolded protein response (UPR) and autophagy have been associated to cellular senescence. Thus, although both signaling pathways play positive roles in the maintenance of a healthy proteostasis and therefore prevent the development of cell senescence, hyperactivation of these pathways under certain conditions, such as chronic stress, can favor several senescence-associated phenotypes [61, 62]. FTIRM allowed us to evaluate biochemical changes in the most important biomolecules. Principal Component Analysis (PCA) in the Fingerprint region (mostly accounting for proteins, DNA and carbohydrates) showed differences among the groups along PC3 (Figure 4G). ‘UVB’ and ‘UVB SA’ groups overlapped and were separated from control group (‘No UVB’). Interestingly, the distribution of ‘UVB SA+EX-527’ group was closer to control. An analysis of the loading plot (Supplementary Figure 6D) shows that most of the differences were due to changes in the Amide I, mostly related to conformational modifications in the proteins. The Amide I deconvolution allowed us to estimate changes in the secondary protein structure, which revealed a global increase of the β-sheet/α-helix ratio in the proteins of these cells upon UVB (Figure 4G, 4H and Supplementary Figure 6D). This ratio increase has been previously linked to increased levels of misfolded proteins [63–66], suggesting that SA prevents the accumulation of misfolded proteins accumulated upon UV. Strikingly, while SA treatment inhibited these levels well below the ratio observed in control cells in the absence of UVB irradiation, treatment with EX-527 completely reverted the effect of SA (Figure 4G, 4H and Supplementary Figure 6D). This suggested that the SIRT1-dependent effect of SA on senescence may be associated with an altered pattern of misfolded and/or aggregated proteins, which pointed to an effect on UPR and/or autophagy. Interestingly, while SA treatment induced upregulation of chaperone BiP (also known as GPR78), a major regulator of cell proteostasis, that effect was partially reverted by EX-527 (Figure 5A, 5B). In agreement with a key role of BiP in SA signaling, siRNA-induced downregulation of BiP partially reverted the anti-senescence effect of SA (Figure 5C and Supplementary Figure 7A). The excess of available BiP protein has been linked with UPR inhibition [67], so we hypothesized that under these conditions SA blocks UPR signaling, and subsequently UPR-induced autophagy. Accordingly, SA treatment induced a decrease in spliced-XBP1, a marker of UPR activity (Figure 5D). Consistently with a direct connection between the effect of SA on BiP and UPR, we detected a SA-dependent decrease in the levels of LC3-II, a well-established marker of autophagy. In agreement with a link with the SA/SIRT1 signaling axis, this effect was mostly reverted by EX-527 treatment (SA 10mM). Interestingly, this EX-527 effect was in turn reversed by increased levels of SA (25 and 50 mM) (Figure 5C and Supplementary Figure 7B). Taken together, these results suggest that the SIRT1-dependent protective effect of SA on senescence is associated with preventing the cellular accumulation of misfolded proteins.

## DISCUSSION

Our work reveals a role for SA in preventing cellular senescence in HDFs and identifies SIRT1 as a key downstream effector of this function (Figure 5D). Our evidences suggest that the signaling axis involving SA/SIRT1 contributes to maintenance of proteostasis. However, at this point we cannot discard that the SA/SIRT1 signaling could prevent senescence through other mechanisms, or that SA may regulate senescence through other SIRT1-independent mechanisms. In fact, our study suggests that SIRT1 is not targeted directly and it’s instead a downstream effector of SA based on several evidences: i) SA does not alter SIRT1 activity *in vitro*; ii) SA does not have a clear effect in UV-induced cell death, DNA damage or genome stability while SIRT1 has important roles in these pathways; and iii) SIRT1 inhibitor EX-527 cannot revert the effect of SA on the expression levels of pro-inflammatory IL-6. This suggests that SA signaling activates SIRT1 activity specifically in this context of senescence, but has no further effect on other SIRT-associated functions. This is also suggested by the different behavior of the other screening candidates, such as resveratrol and quercetin, which promoted sirtuin activity but had different effect on senescence markers. Nevertheless, the lack of effect of SA on genome stability may not be the case in other tissues, given that SA protects against oxidative stress-induced damage in hepatocytes and neuroblastoma cells [35, 68]. Despite all these evidences, the mechanism of SIRT1 activation by SA signaling remains unaddressed. One interesting possibility is that the observed activating effect of shikimic acid may involve SIRT1 phosphorylation and subsequent activation. Thus, SIRT1 activators JNK2 and AMPK, two key stress-dependent kinases, have been functionally linked with protective effect upon ER stress and autophagy, which may involve SIRT1 activation [69, 70]. Although not present in animals, in many organisms such as plants, bacteria and fungi, SA is a key metabolic intermediate at the crossroads of several essential metabolic pathways, such as glycolysis and the biosynthesis of aromatic aminoacids and metabolites like coenzyme Q and vitamin K [71]. Moreover, SA is commonly used in many applications, given that it has an antibacterial and antifungal activity and is widely used in biomedicine as a precursor in the chemical synthesis of several anticancer and antiviral compounds [72]. In the case of skin, SA is used to treat bacterial infections and acne and is known to induce skin exfoliation. The important metabolic role of SA in non-animal organisms suggests that the effect of SA in animals may take place through other conserved pathways associated to SA signaling in these non-animal organisms. In this sense, it would be relevant to determine whether SA has an anti-senescent role also in these non-animal organisms. Moreover, we cannot discard that the observed anti-senescent effect in human cells is tissue-specific and that it may not have the same protective effect beyond skin. Further studies will be required to define how conserved is the role of SA in this context and the mammalian pathways involved in this protective role.

The role of this compound as a metabolic intermediate in other organisms and its high turnover in the cell probably explain the very low toxicity seen in our experiments, and the concentration levels required to protect senescence and induce SIRT1 activity. This is a similar case to that of nicotinamide, a well-established sirtuin inhibitor and key metabolic intermediate that is used in cellular concentrations of 5-10 mM in a variety of human cell types, from neural to stem cells [73].

The lack of specific senescence markers is a current challenge in the field and there is a clear requirement for novel simple combinations in each specific context that may allow easy and efficient senescence detection. Our studies imply that the SA-β-Gal/*HAS2* combination is a *bona fide* marker of skin cell senescence, in contrast to other factors, such as p21 or p16. In the case of skin, several reports already indicated that p21 or p16 levels might not be reliable markers of senescence, as the pathways activated during senescence are influenced by the stress stimulus and cell type [74, 75]. This probably reflects a specific feature of UV-induced senescence in skin cells.

Another interesting finding of our work is the effect of SA in cell proteostasis, UPR and autophagy. Previous results have portrayed the complex relationship between UPR, autophagy and skin senescence, and it is generally accepted that abnormal hyperactivation of UPR and autophagy promotes the development of cellular senescence [61, 62]. UPR sustained signaling has been shown to activate autophagy and to promote cell senescence at different levels [62]. We hypothesize that under senescence, SA induces BiP upregulation to promote the recovery of a healthy proteostasis, which in turn promotes a block of UPR receptors by available BiP [67], and a concomitant decrease in the signaling of UPR and associated autophagy. In particular, our results indicate that the addition of SA on HDFs undergoing UV-induced senescence impairs the formation of spliced XBP1, a UPR-dependent transcription factor previously shown to promote autophagy under ER stress [76].

Our findings together with the low toxicity of SA suggest that it has considerable potential in the treatment against skin senescence and aging induced by UV irradiation. Future studies should confirm whether this is the case and define the precise contribution of SIRT1 in this context.

## MATERIALS AND METHODS

### Cell culture

Human dermal fibroblasts (HDFs) (Promocell, Germany) from 40-year old Caucasian women were used as the *in vitro* model for compound viability and efficacy screening. Cells were cultured in Dulbecco's modified eagle medium (DMEM) low glucose (Biowest, France) supplemented with 10% fetal bovine serum (FBS) (Capricorn Scientific, Germany) and 10 U/mL penicillin/streptomycin (Biowest, France) in a humidified incubator at 37° C and 5% CO2. For cell passage, cells at 80% confluence were trypsinized and plated at 60% confluence. Cells between passage 13 and 18 were used for the assays. For the SIRT1 *in vitro* assay, HEK293 cells (ATCC, USA), used to transfect and immunoprecipitate SIRT1 protein, were grown in DMEM high glucose (Thermo Fisher Scientific, USA), supplemented with 10% FBS in a humidified incubator at 37° C and 5% CO2. The following experiments were all performed in triplicate.

### Cell viability assays, SiRNAs, compound treatments and senescence

HDFs were plated in 100x20 mm2 cell culture plates and after 24h, fresh cell culture medium containing the compounds was added. After 24h, cells were detached with cell scrapers, harvested, and processed for qPCR or western blot.

The protocol for senescence induction using UVB irradiation is described in the Supplementary Methods. SA-β-Gal staining, WST-1, sulforhodamine B (SRB) and trypan blue assays were performed as previously described [77–80]. For the siRNA experiments, the protocol for senescence induction using UVB irradiation was the same as in the Supplementary Methods. Specific siRNA (Horizon discovery, UK) for SIRT1, SIRT2, SIRT6, BiP or scramble siRNA were incubated for 6 hours before each irradiation step.

### Gene expression quantification by qPCR

RNA was extracted from cell pellets using the Total RNA Purification Kit (Norgen, Canada), according to the manufacturer’s protocol. Extracted RNA was quantified using a NanoDrop® ND-1000 UV-Vis Spectrophotometer (Thermo Fisher Scientific, USA) and 300 ng of RNA were retrotranscribed to cDNA using the PrimeScriptTM RT reagent kit (Takara Bio Inc., Japan). cDNA was diluted 1/10 in ultrapure water and qPCR mix was prepared by adding cDNA (12 ng), SYBR green supermix (Bio Rad, USA), forward and reverse primers (0.2μM). The primers and their respective sequences are listed in Supplementary Table 1. qPCR protocol included one step at 95° C for 30 s, 40 cycles at 95° C for 10s, and 60° C for 1min. The results were analyzed with Bio-Rad CFX Manager software. BACT was used as a housekeeping gene to normalize gene expression.

### Protein level quantification by western blot

Protein levels were analyzed as previously described [81]. Cells were directly lysed in 1x Laemmli Buffer (Bio-Rad, USA), incubated at 95° C and centrifuged. Supernatant was subjected to SDS-polyacrylamide gel electrophoresis, and successive steps were performed as described [81]. The list of antibodies used is included in Supplementary Table 2.

### Flow cytometry

Fluorescence-activated cell sorting (FACS) analyses of the cell cycle and apoptosis were carried out using, respectively, a Propidium Iodide Flow Cytometry Kit (Abcam, UK), and an Annexin V-FITC Apoptosis Staining/Detection Kit (Abcam, UK), following the manufacturer’s instructions.

### γH2AX immunofluorescence assay

The staining and detection of γH2AX foci by immunofluorescence was performed as previously described [82]. Slides were analyzed by confocal microscopy using a Confocal Leica SP5 microscope (Leica Microsystems) with a 63x objective.

### *In vitro* assay for SIRT1 activity

The *in vitro* SIRT1 deacetylation assay was performed as previously described [83]. Briefly, 500ng of the purified SIRT1-FLAG from HEK293F cells were incubated with 1200ng of hyperacetylated histones (purified by acid extraction) or 500ng of purified FLAG-p53 (anti-FLAG resin, Sigma-Aldrich) in 30μl of deacetylation buffer (50 mM-Tris pH7.8, 4mM MgCl_2_, 0.2mM DTT) for 15min at 37° C in the presence or absence of 1 mM NAD+ and/or SA at 0-10mM (dissolved in Tris 50mM pH 7.8). The reaction was stopped by adding Laemmli sample buffer. Deacetylation was monitored by quantification of H3K9ac/H3, H4K16ac/H4 and p53K382ac/p53 levels.

### Fourier transform infrared microspectroscopy (FTIRM) analysis

FTIRM measurements were performed at the MIRAS beamline of ALBA Synchrotron using the internal (Globar®) source. The Hyperion 3000 microscope (Bruker) coupled to a Vertex 70 spectrometer and equipped with liquid Nitrogen cooled Mercury Cadmium Telluride (MCT) detector was used for the measurements. The spectra of individual cells (more than 100 per sample) were collected using a single mask aperture of 20×20 μm2. The infrared spectra were acquired in transmission mode in the 4000–900 cm−1 mid-infrared range at a spectral resolution of 4 cm−1 with 512 co-added scans per spectrum. Principal Component Analysis (PCA) was performed on vector normalized second derivative spectra (Savitzky-Golay algorithm, 11 smoothing points) using the Orange toolbox (Bioinformatics Lab) [63, 84]. The structure content of the amide I peak (1710-1598 cm−1) was determined by band deconvolution using Gaussian band fitting profiles (Origin 7.0 software). The position and number of peaks, which were assigned to specific types of secondary structures [85], were determined using the second derivative of the spectra.

### Data availability statement

The data supporting the findings of this study are included in the article and the supplementary information. Additional data or material related to this paper is available upon request to the authors.

## Supplementary Material

Supplementary Methods

Supplementary Figures

Supplementary Tables
