# Supplementary material for: Shikimic acid protects skin cells from UV-induced senescence through activation of the NAD+-dependent deacetylase SIRT1
Source: Aging (Albany NY). 2021 Apr 26;13(9):12308–33. doi: 10.18632/aging.203010 (PMC8148468; doi:10.18632/aging.203010)
Supplement: Supplementary Methods [file aging-13-203010-s001.pdf]

SUPPLEMENTARY METHODS

Senescence induction by UVB irradiation

HDF were plated at a density of 6×104 cells/plate in 6-well plates. After 24h, culture medium was removed and replaced with fresh cell culture medium containing the compounds at different concentrations. After 24h, cell culture medium was removed, and cells were irradiated at 25 mJ/cm^2 of UVB light (312 nm) in a Bio-LINK Crosslinker BLX-312/365 (Witec ag) in a small amount of PBS. PBS was then removed and fresh cell culture medium with the compound was added to each well. After 3 days, cells were irradiated again under the same conditions. After 2 days, cells were processed according to the qPCR, β-Gal staining, apoptosis, cell cycle or immunofluorescence protocols.

For the non-irradiation control conditions, HDF were plated at a density of 6×104 cells/plate in 6-well plates. After 24h, culture medium was removed and replaced by fresh cell culture medium containing the compounds at different concentrations. After 24h, cells were trypsinized and reseeded at 6×104 cells/plate in fresh cell culture medium containing the compound in 6-well plates. After 3 days, cells were trypsinized again and reseeded at 8×104 cells/plate in fresh cell culture medium containing the compound in 6-well plates. After 2 days, cells were processed according to the qPCR, β-Gal staining, apoptosis, cell cycle or immunofluorescence protocols.
