# Supplementary material for: Shikimic acid protects skin cells from UV-induced senescence through activation of the NAD+-dependent deacetylase SIRT1
Source: Aging (Albany NY). 2021 Apr 26;13(9):12308–33. doi: 10.18632/aging.203010 (PMC8148468; doi:10.18632/aging.203010)
Supplement: Supplementary Figures [file aging-13-203010-s002.pdf]

SUPPLEMENTARY FIGURES

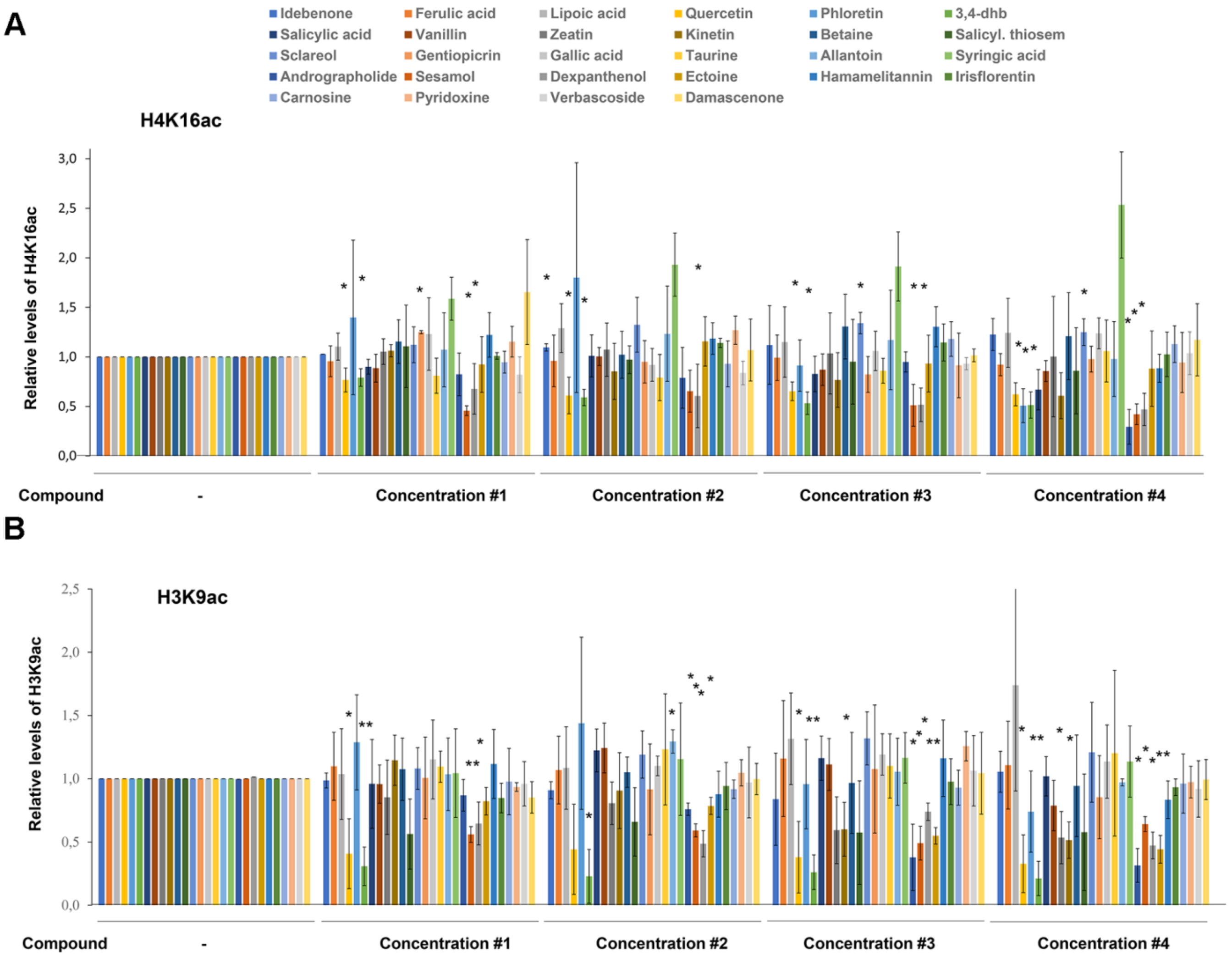

Supplementary Figure 1. Sirtuin activity assay results. (**A**) Quantification of H4K16ac levels and (**B**) H3K9ac levels from (n=3) Western-blots as in Figure cells treated with the following compounds during 24h: Idebenone (0.5, 1, 2.5, 5 µM), Ferulic acid (50, 100, 200, 500 µM), Lipoic acid (50, 100, 200, 500 µM), Quercetin (50, 100, 200, 250 µM), Phloretin (10, 25, 50, 75 µM), 3,4-dihydroxybenzaldehyde (3,4-dhb, 0.5, 0.75, 1, 2 mM), Salicylic acid (0.5, 0.75, 1, 2.5 mM), Vanillin (0.5, 0.75, 1, 2.5 mM), Zeatin (0.25, 0.5, 0.75, 1 mM), Kinetin (0.5, 0.75, 1, 2 mM), Betaine (5, 10, 25, 50 mM), Salicylaldehyde thiosemicarbazone (50, 100, 250, 500 µM), Sclareol (5, 10, 20, 40 µM), Gentiopicrin (250, 500, 750, 1000 µM), Gallic acid (25, 50, 75, 100 µM), Taurine (5, 10, 25, 50 mM), Allantoin (5, 10, 25, 50 mM), Syringic acid (0.5, 1, 2.5, 5 mM), Andrographolide (1, 5, 10, 20 µM), Sesamol (100, 250, 500, 1000 µM), Dexpanthenol (10, 25, 50, 100 mM), Ectoine (50, 100, 250, 300 mM), Hamamelitannin (25, 50, 75, 100 µM), Irisflorentin (10, 25, 50, 75 µM), Carnosine (1, 5, 10, 25 mM), Pyridoxine (0.5, 1, 5, 10 mM), Verbascoside (10, 25, 50, 75 µM) and Damascenone (1, 10, 25, 50 µM). The data is grouped in 5 groups including no compound (-) and the four concentrations indicated above for each compound. H4K16 and H3K9 acetylation levels were normalized to H4 and H3 respectively. For each drug all values are represented relative to the condition without compound. Student T-test were performed comparing these values with no compound conditions. *$p<0.05$, **$p<0.01$, ***$p<0.001$.

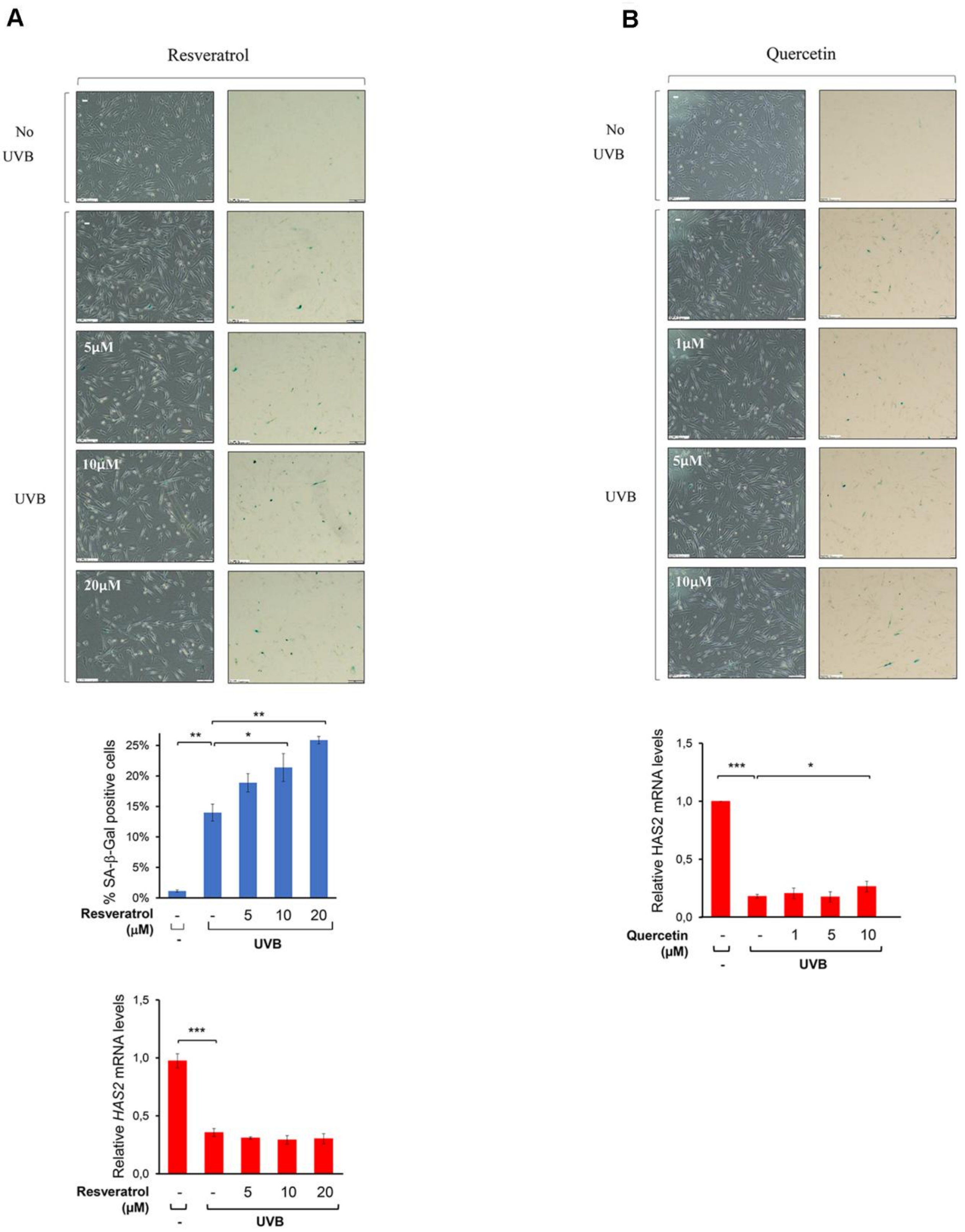

Supplementary Figure 2. Senescence assay results from cells treated with (**A**) Resveratrol or (**B**) Quercetin, including SA-β-Gal staining (phase-contrast image in the left column and bright-field image in the right column) and relative *HAS2* mRNA levels (n=3) compared with control conditions without UVB treatment in absence of shikimic acid. In the case of resveratrol, a quantification (n=3) of SA-β-Gal is also included. Student T-test, *p<0.05, **p<0.01, ***p<0.001.

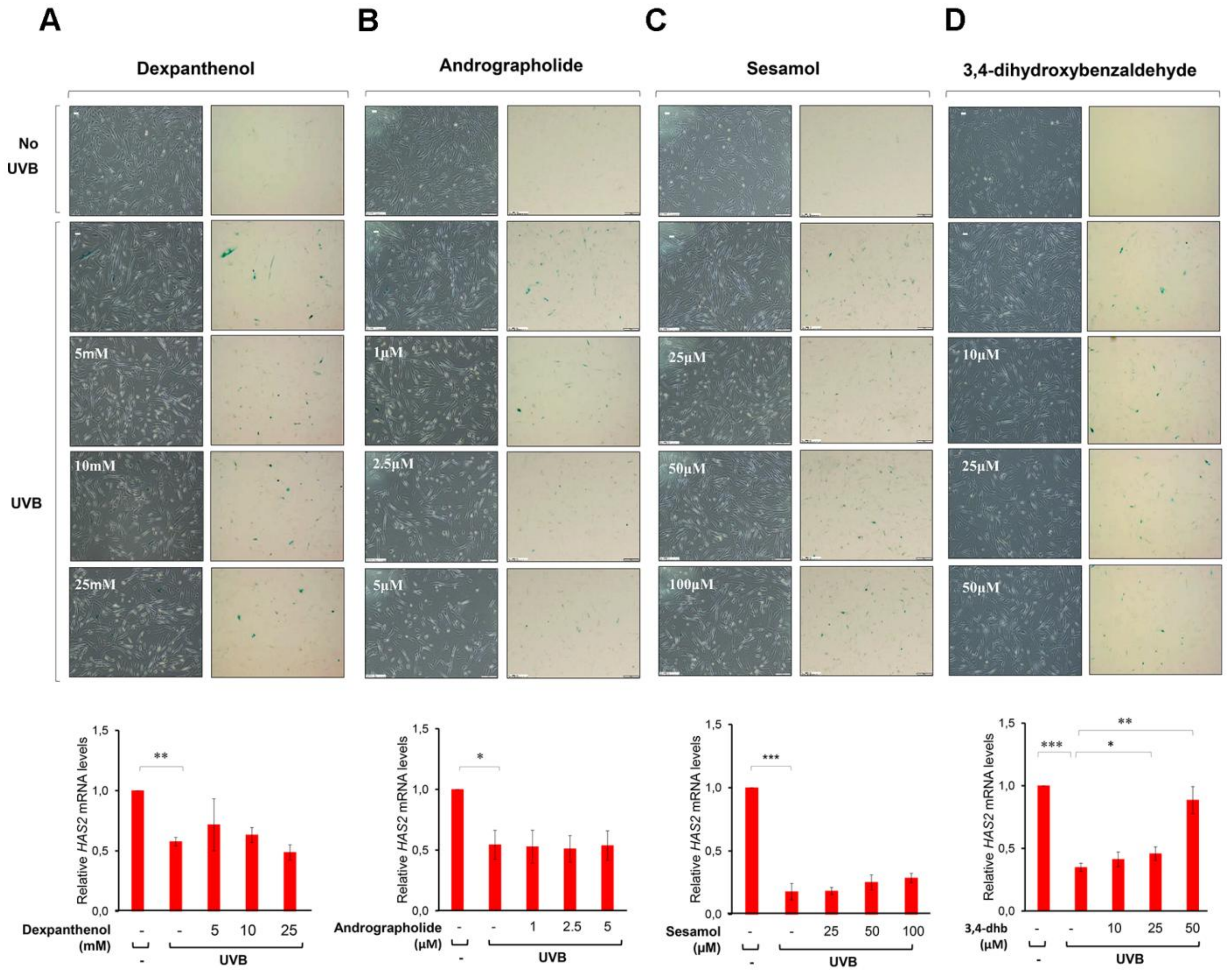

Supplementary Figure 3. Senescence assay results from cells treated with (**A**) Dexpanthenol, (**B**) Andrographolide, (**C**) Sesamol or (**D**) 3,4-dihydroxybenzaldehyde, including SA-β-Gal staining (phase-contrast image in the left column and bright-field image in the right column) and relative *HAS2* mRNA levels (n=3) compared with control conditions without UVB treatment in absence of shikimic acid. Student T-test, *p<0.05, **p<0.01, ***p<0.001.

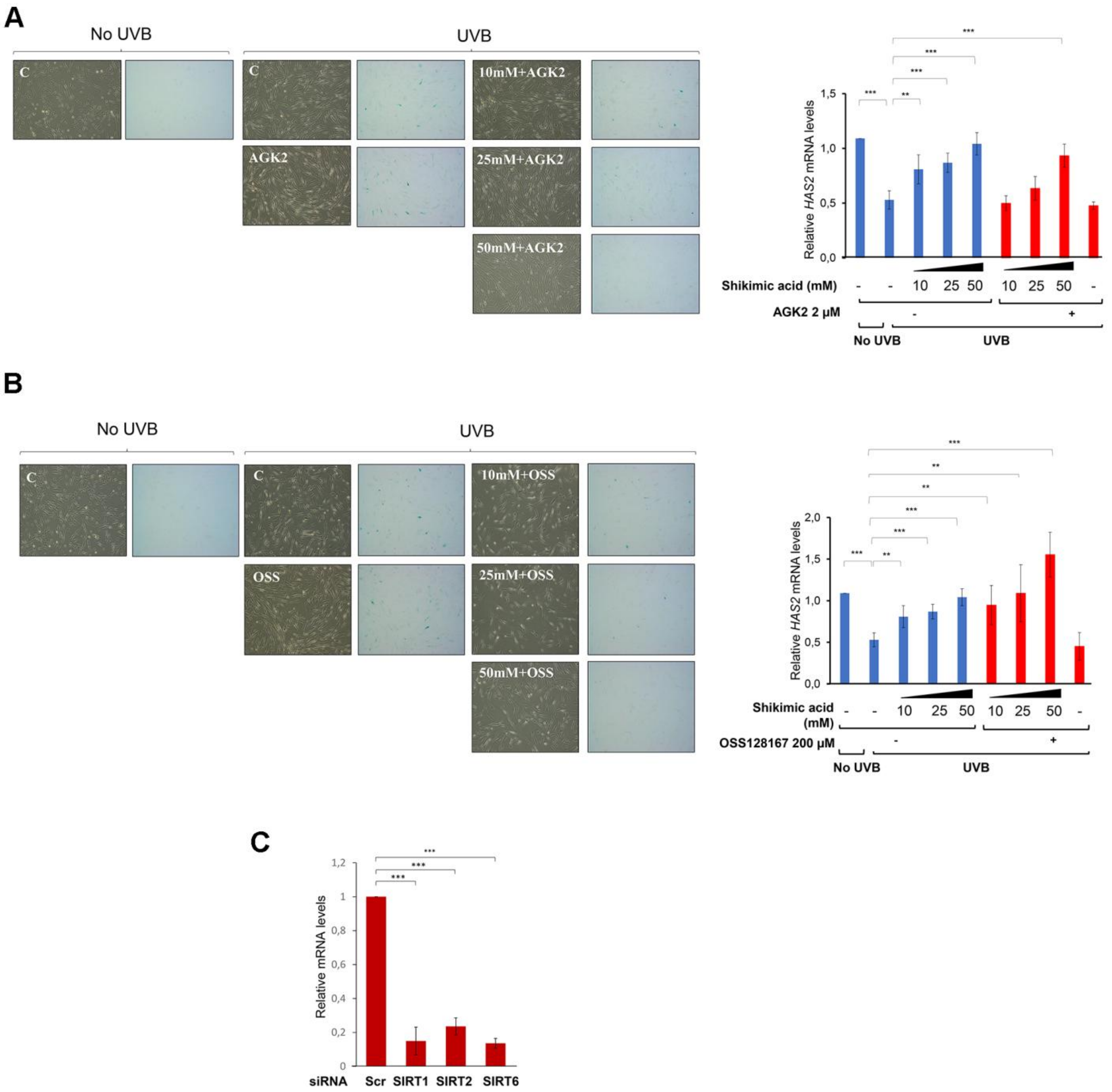

Supplementary Figure 4. Senescence assay results from cells treated with or without Shikimic acid (10, 25 and 50 mM) plus (**A**) AGK2 2 µM or (**B**) OSS128167 200 µM, including SA-β-Gal staining (phase-contrast image in the left column and bright-field image in the right column) and relative *HAS2* mRNA levels (n=3) compared with control conditions without UVB treatment in absence of shikimic acid. Student T-test, *p<0.05, **p<0.01, ***p<0.001. (**C**) mRNA levels of SIRT1, SIRT2, SIRT6 in the siRNA-mediated downregulation experiments shown in Figure 3E. Scr: Scramble siRNA. Student T-test, ***p<0.001.

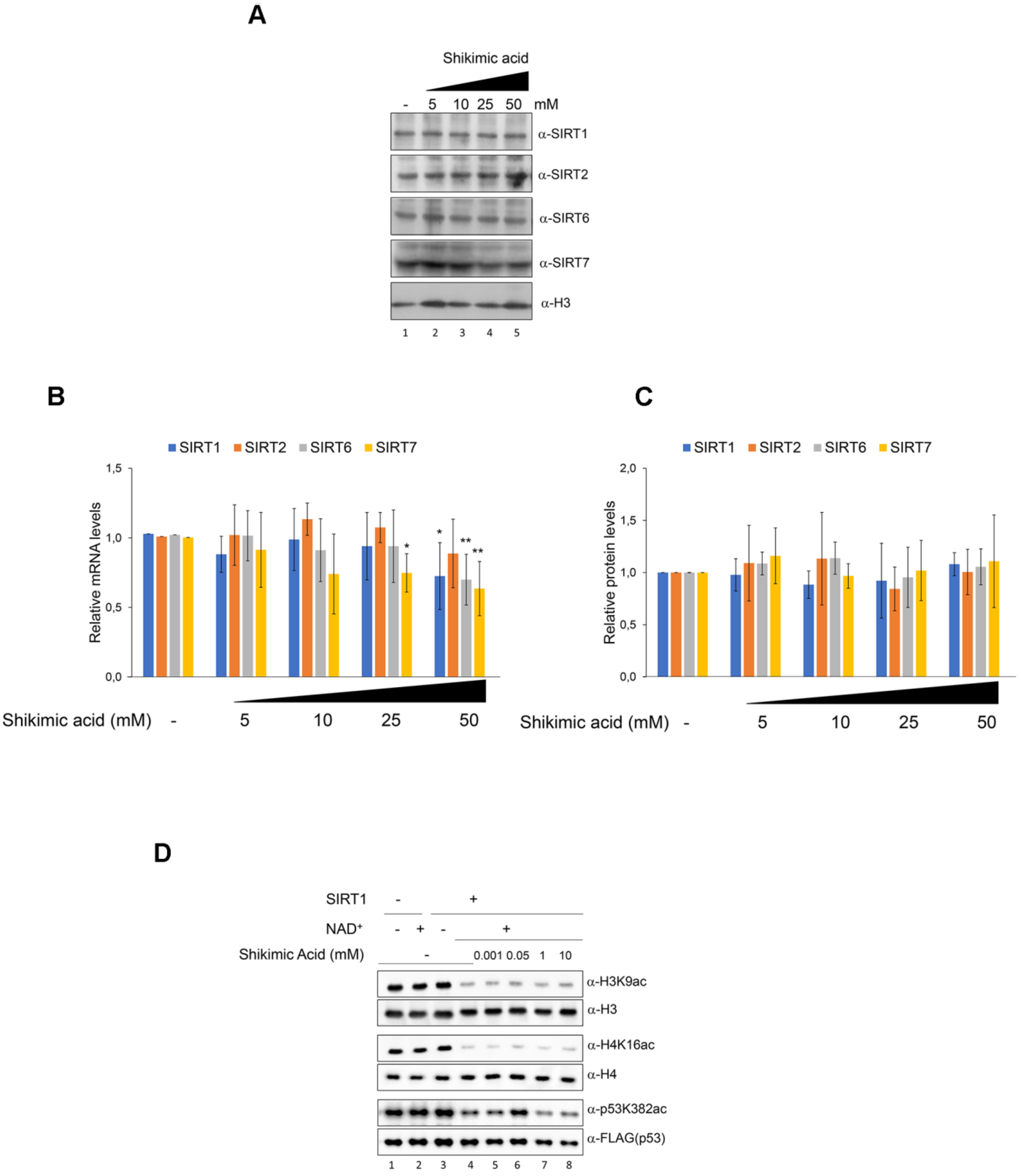

Supplementary Figure 5. (**A**) Western Blot analysis of SIRT1, SIRT2, SIRT6 and SIRT7 protein levels in non-treated cells and cells treated with Shikimic acid (5, 10, 25 and 50 mM). (**B**) Relative mRNA levels and (**C**) relative protein levels quantification (n=3) of SIRT1, SIRT2, SIRT6 and SIRT7 in HDF treated with Shikimic acid at the indicated doses for 24h. (**D**) A representative Western-Blot of SIRT1 *in vitro* activity assay at the indicated doses of Shikimic acid and quantified in Figure 3H. FLAG-p53 was affinity purified with FLAG-resin and monitored with anti-FLAG anti body. The levels of H3K9 ac/H3, H4K16 ac /H4 and p53K382 ac/p53 are shown. Student T-test, *p<0.05, **p<0.01, ***p<0.001.

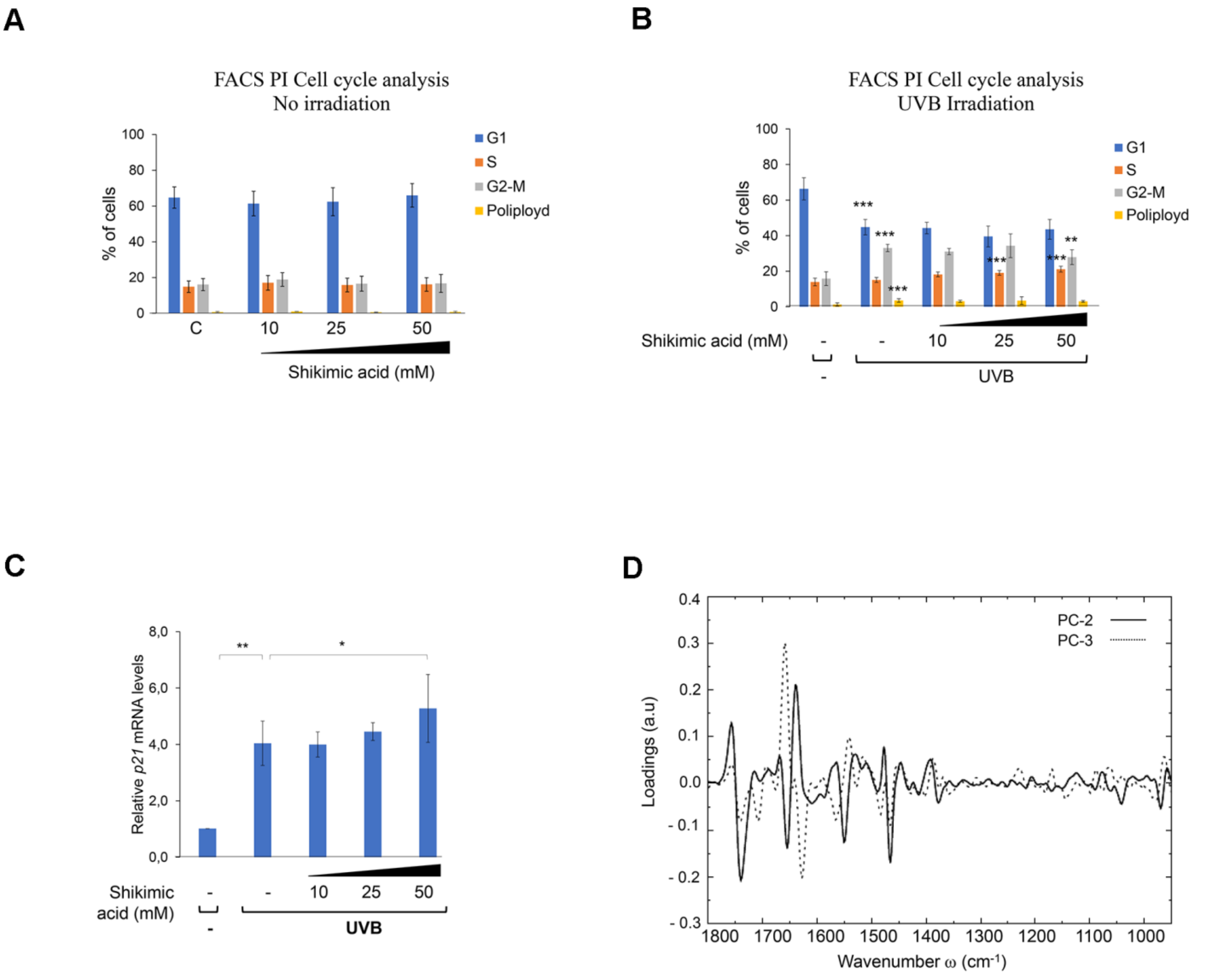

Supplementary Figure 6. Quantification (n=3) of the percentage of cells in each cell cycle phase and poliploydies in (**A**) non-irradiated cells treated with Shikimic acid at the indicated doses and (**B**) UVB-irradiated cells treated with Shikimic acid at the indicated doses, following the senescence induction method described in Figure 2A. A representative experiment of n=3 experiments is shown in Figure 4D. (**C**) Relative *p21* mRNA levels (n=3) in UVB-irradiated cells treated with Shikimic acid at the indicated doses, following the senescence induction method described in Figure 2A. (**D**) Second (PC-2) and third (PC-3) principal components in the fingerprint region (1800–950 cm−1) from the samples mentioned in Figure 4G. Student T-test, *p<0.05, **p<0.01, ***p<0.001.

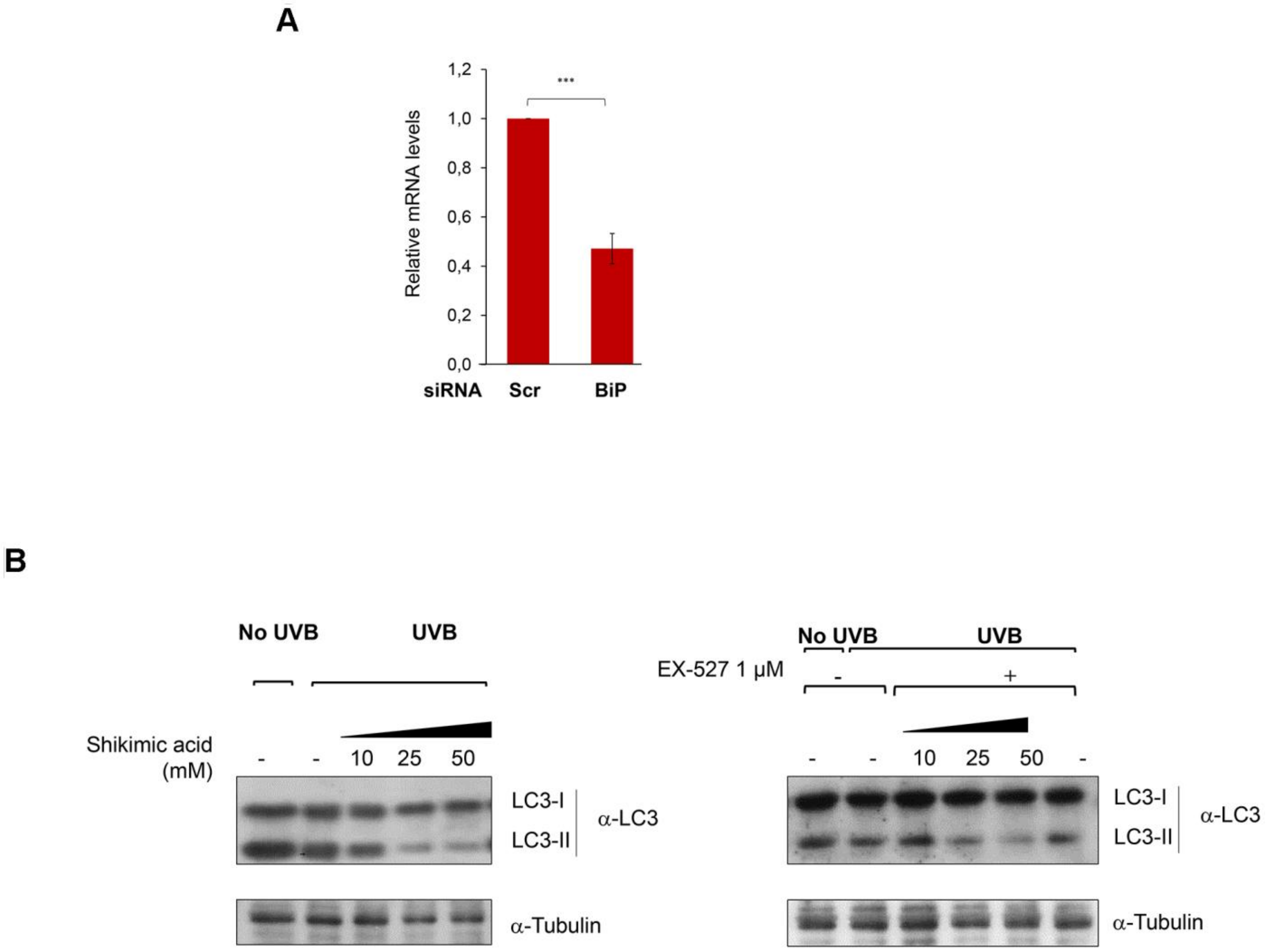

Supplementary Figure 7. (**A**) mRNA levels of BiP in the siRNA-mediated downregulation experiments shown in Figure 5C. Scr: Scramble siRNA. (**B**) non-irradiated A representative Western-Blot of LC3-I/II and Tubulin of the quantifications shown in Figure 5E. Student T-test, ***p<0.001.
