# Supplementary material for: Shikimic acid protects skin cells from UV-induced senescence through activation of the NAD+-dependent deacetylase SIRT1
Source: Aging (Albany NY). 2021 Apr 26;13(9):12308–33. doi: 10.18632/aging.203010 (PMC8148468; doi:10.18632/aging.203010)
Supplement: Supplementary Tables [file aging-13-203010-s003.pdf]

SUPPLEMENTARY TABLES

Supplementary Table 1. List of primers for qPCR analysis.

Gene	Forward primer	Reverse primer
β-actin	5′-ACTGGAACGGTGAAGGTGACA-3′	5′-ATGGCAAGGGACTTCCTGTAAC-3′
HAS2	Bio Rad	Bio Rad
IL-6	Bio Rad	Bio Rad
p16	Bio Rad	Bio Rad
p21	5′-CTGGAGACTCTCAGGGTCGAA-3′	5′-CCAGGACTGCAGGCTTCC T-3′
SIRT1	5’-TGGGTACCGAGATAACCTTCT-3’	5’-TGTTCGAGGATCTGTGCCAA-3’
SIRT2	Bio Rad	Bio Rad
SIRT6	5’-GCAGTCTTCCAGTGTGGTGT-3’	5’-AAGGTGGTGTCGAACTTGGG-3’
SIRT7	5’-ACTTGGTCGTCTACACAGGC-3’	5’-CAGCACTAACGCTTCTCCCT-3’
sXBP1	5’-CTGAGTCCGCAGCAGGTGCA-3’	5’-GGTCCAAGTTGTCCAGAATGCCCAA-3’
uXBP1	5’-ACTCAGACTACGTGCACCTC-3’	5’-GTCAATACCGCCAGAATCCA-3’

Supplementary Table 2. List of antibodies for western blot analysis.

Antibody	Dilution	Purchase information
Anti-H3K9ac (rabbit)	1:1000	Cell Signaling (#9649)
Anti-H3 (rabbit)	1:10000	Cell Signaling (#9715)
Anti-H4K16ac (rabbit)	1:1000	Millipore (#07-329)
Anti-H4 (rabbit)	1:10000	Abcam (#ab10158)
Anti-SIRT1 (mouse)	1:1000	Abcam (#ab7343)
Anti-SIRT2 (rabbit)	1:1000	Abcam (#ab51023)
Anti-SIRT6 (rabbit)	1:1000	Abcam (#ab62739)
Anti-SIRT7 (rabbit)	1:1000	Cell Signaling (#5360S)
Anti-Tubulin (mouse)	1:20000	Sigma-Aldrich (#T5168)
Anti-LC3B (rabbit)	1:500	Cell Signaling (#2775S)
Anti-p62 (rabbit)	1:1000	Abcam (#ab91526)
Anti-BiP (rabbit)	1:1000	Cell Signaling (#3177S)
Anti-IgG rabbit (donkey)	1:3000	Tebu bio (#611-7302)
Anti-IgG mouse (goat)	1:10000	Sigma-Aldrich (#A9917)
Anti-p53K382ac (rabbit)	1:1000	Abcam (ab75754)
Anti-FLAG (rabbit)	1:1000	Sigma-Aldrich (#F7425)
